# Abdominal Cocoon Presenting As Subacute Intestinal Obstruction

**DOI:** 10.7759/cureus.65493

**Published:** 2024-07-27

**Authors:** Kausar A Fakih, Sania Khalifa, Mansha Singh, Sandhya Iyer

**Affiliations:** 1 General Surgery, Lokmanya Tilak Municipal Medical College and General Hospital, Mumbai, IND; 2 General Surgery, Grant Government Medical College and Sir JJ Group of Hospitals, Mumbai, IND

**Keywords:** midgut malrotation, encapsulating membrane, peritoneal dialysis, intestinal obstruction, abdominal cocoon, sclerosing encapsulating peritonitis

## Abstract

An abdominal cocoon is the formation of a fibro-collagenous membrane that surrounds the small bowel like the larval cocoon. We present a rare case of abdominal cocoon presenting as a subacute intestinal obstruction in a patient with partial midgut malrotation. The case was confirmed with CT after initial clinical examination. The patient underwent laparotomy with excision of the sac and adhesiolysis followed by an uneventful recovery. Since idiopathic abdominal cocoon is rare and so is its manifestation as subacute intestinal obstruction, we wish to add this case to the current scientific literature.

## Introduction

Abdominal cocoon (primary idiopathic sclerosing encapsulating peritonitis (SEP)) is a condition in which a thick fibrotic membrane encloses a part or entire small bowel, and rarely, it may extend to include other abdominal viscera. It was first termed “peritonitis chronica fibrosa incapsulata” by Owtschinnikow in 1907 and later abdominal cocoon by Foo in 1978 [1]. The exact prevalence of abdominal cocoon syndrome is unknown [2]. These patients present with non-specific symptoms like chronic abdominal pain, malnutrition, weight loss, nausea, vomiting, or acute abdomen [3]. Common causes of small bowel obstruction in emergencies are adhesions, obstructed hernias, abdominal tuberculosis, and malignancy; only 6% have unusual causes, and abdominal cocoon contributes to a small part of it [4]. Diagnosis and treatment can be a challenge due to vague symptoms.

## Case presentation

A 40-year-old male presented to a surgical emergency with a seven-day history of abdominal distension, constipation, dull pain all over the abdomen, and multiple episodes of vomiting. The patient did not have nausea or anorexia. No similar complaints in the past. He had pulmonary tuberculosis 30 years ago, for which treatment was completed.

**Figure 1:**
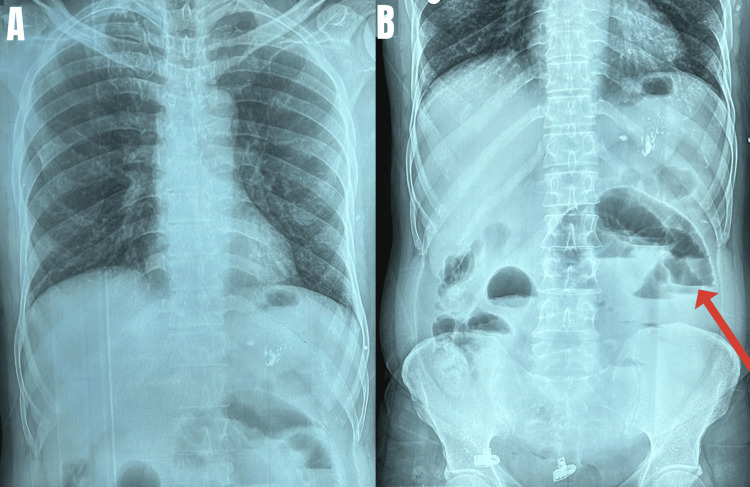
(A) Chest X-ray; (B) abdomen X-ray showing multiple air-fluid levels (red arrow) suggestive of intestinal obstruction

On examination, the patient was vitally stable and the abdomen was soft on palpation with mild distension and no associated tenderness or guarding. Bowel sounds were normal. Routine blood investigations (complete blood count, serum electrolytes, renal function test, and liver function test) were within normal limits (Table 1). The abdominal X-ray showed few air-fluid levels (Figure 1). Sonography was normal. The patient was investigated further with a CT scan (Table 2).

**Table 1:** Laboratory tests SGOT: serum glutamic oxaloacetic transaminase; SGPT: serum glutamic pyruvic transaminase; BUN: blood urea nitrogen; INR: international normalized ratio

Laboratory test	Patient’s value	Reference range
Hemoglobin	11	9-15 g/dl
White blood count	12000	5000-12000/cumm
Platelets	330000	150000-500000/cumm
Total bilirubin	1.1	0.1-1.2 mg/dl
SGOT/SGPT	20/30	20-60 IU/L
Total proteins	8.5	6.2-8 gm/dl
Albumin	5	3.5-5.5 g/dl
Globulin	3.5	2.3-3.5 g/dl
BUN	10	6-20 mg/dl
Creatinine	0.7	0.7-1.2 mg/dl
Calcium	10	8-10.5 mg/dl
Sodium	140	136-145 mg/dl
Potassium	4.2	3.5-5.2 mg/dl
Prothrombin time	10	9.6-11.7 sec
INR	1.0	0.9-1.1 sec

**Table 2:** CT scan was suggestive of cocoon formation with partial midgut rotation with features of subacute intestinal obstruction

	CT Findings
1.	A thin hypodense membrane encasing jejunal and proximal ileal loops forming a sac-like structure in the abdomen, with the entry point of the sac being in the epigastric region and the exit point in the right iliac fossa.
2.	Few intervening bowel loops in the sac showed mild luminal dilatation however contrast was seen reaching the descending colon.
3.	Mild ascites with mild mesenteric fat stranding.
4.	Mildly enhancing long segment wall thickening in a proximal jejunal and a mid-ileal loop.
5.	Superior mesenteric vein seen anterior to the superior mesenteric artery; Caecum was seen in the right iliac fossa and duodenojejunal (DJ) flexure was seen right of the midline suggestive of partial midgut malrotation.

The above features were suggestive of abdominal cocoon formation with possible closed-loop obstruction. On further discussion with the radiologist, tuberculosis was considered a differential. The possibility of an internal hernia was considered less likely since entry and exit into the sac-like structure were wide apart.

**Figure 2:**
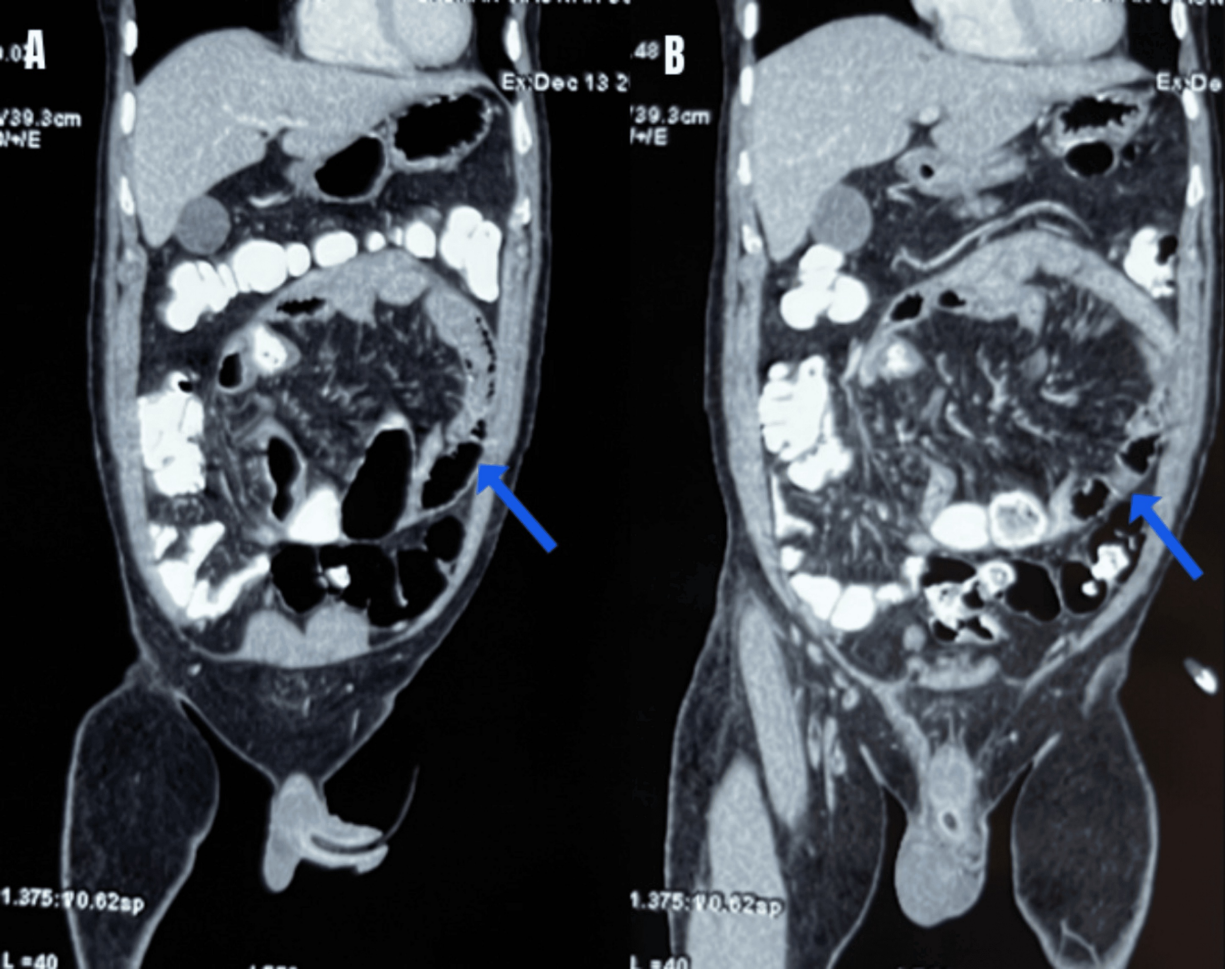
CT abdomen in the coronal plane (A and B) showing membrane (blue arrow) surrounding small bowel loops suggestive of cocoon formation

**Figure 3:**
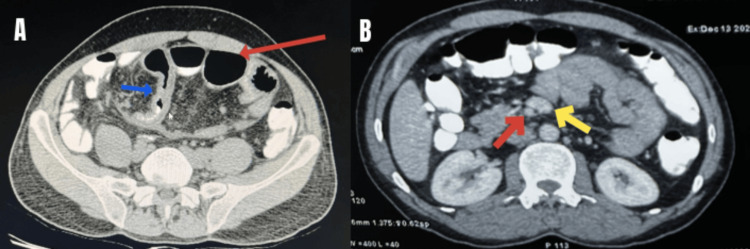
CT abdomen in the axial plane (A) showing dilated bowel loops (red arrow) and thickened bowel loops (blue arrow) in the cocoon (B) superior mesenteric artery (red arrow) seen to the right of the superior mesenteric vein (yellow arrow) suggestive of partial malrotation

Due to persistent pain in the abdomen and CT findings (Figures 2-3) of closed-loop obstruction, it was decided to go ahead with surgery.

Operative findings

**Figure 4:**
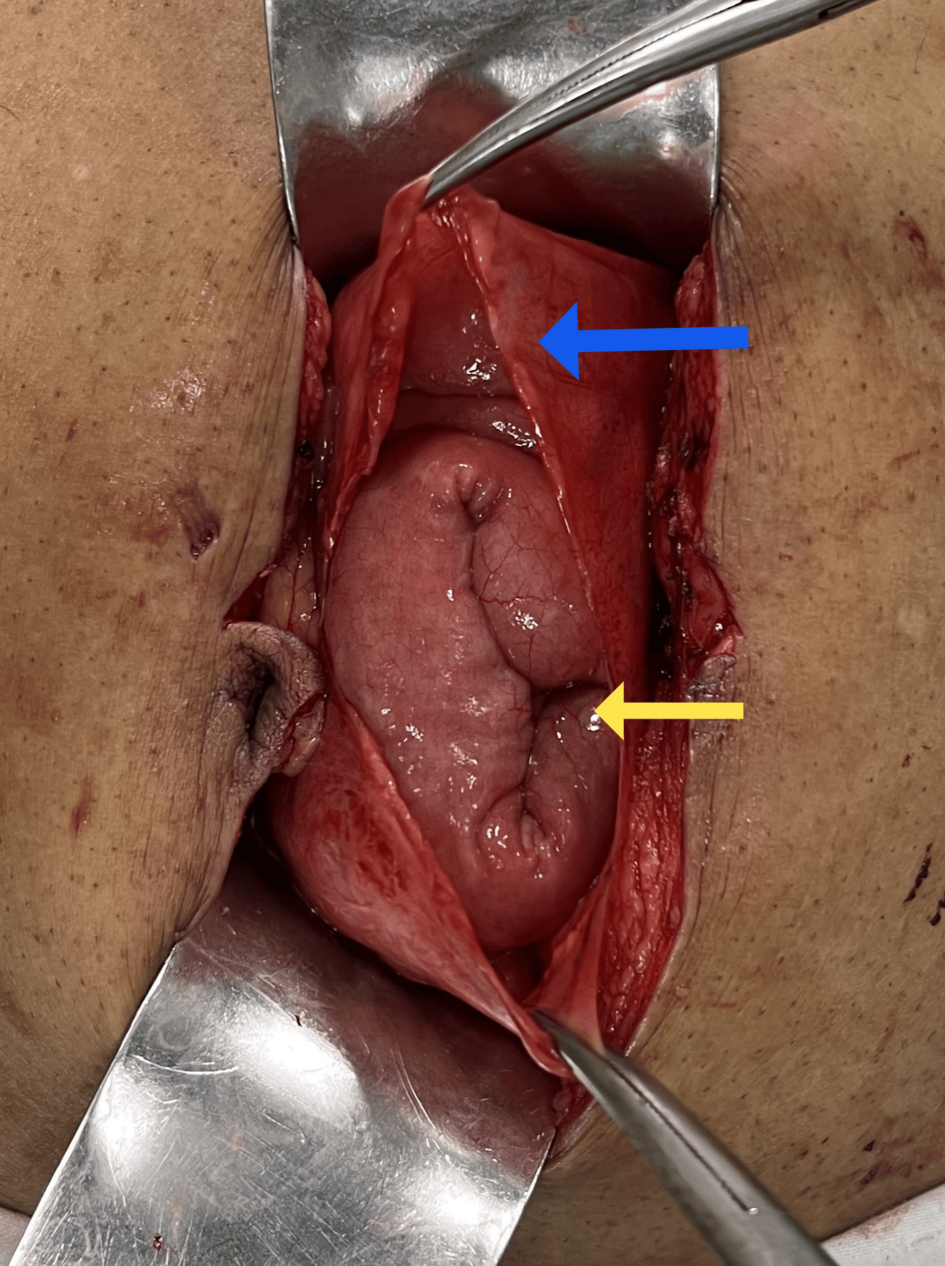
Intra-operative image showing membrane (blue arrow) enclosing small bowel loops (yellow arrow)

**Figure 5:**
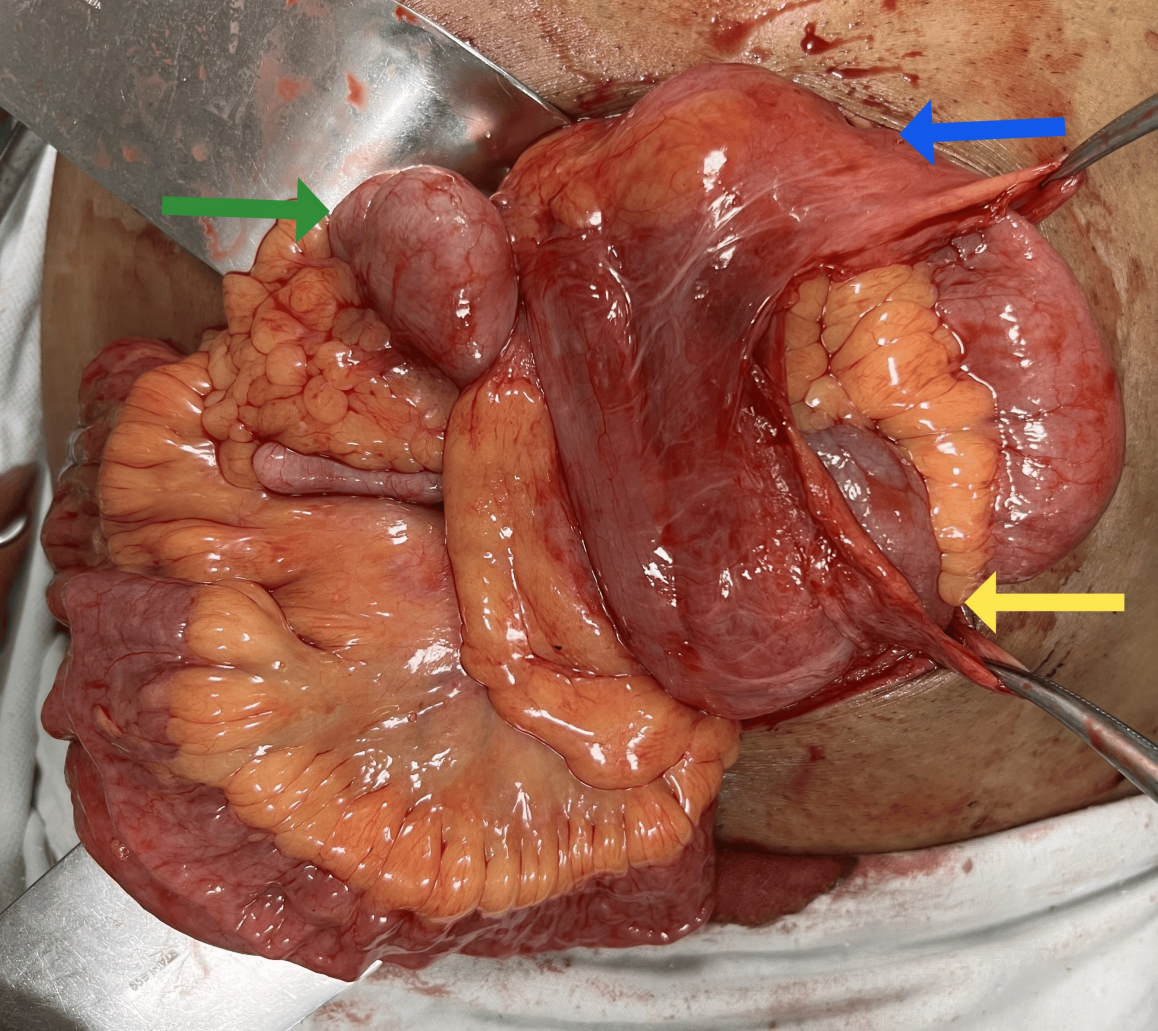
Intra-operative image showing membrane (blue arrow) with enclosed small bowel loops (yellow arrow). A freely mobile caecum (green arrow) along with distal ileal loops is seen lying outside the membrane

**Figure 6:**
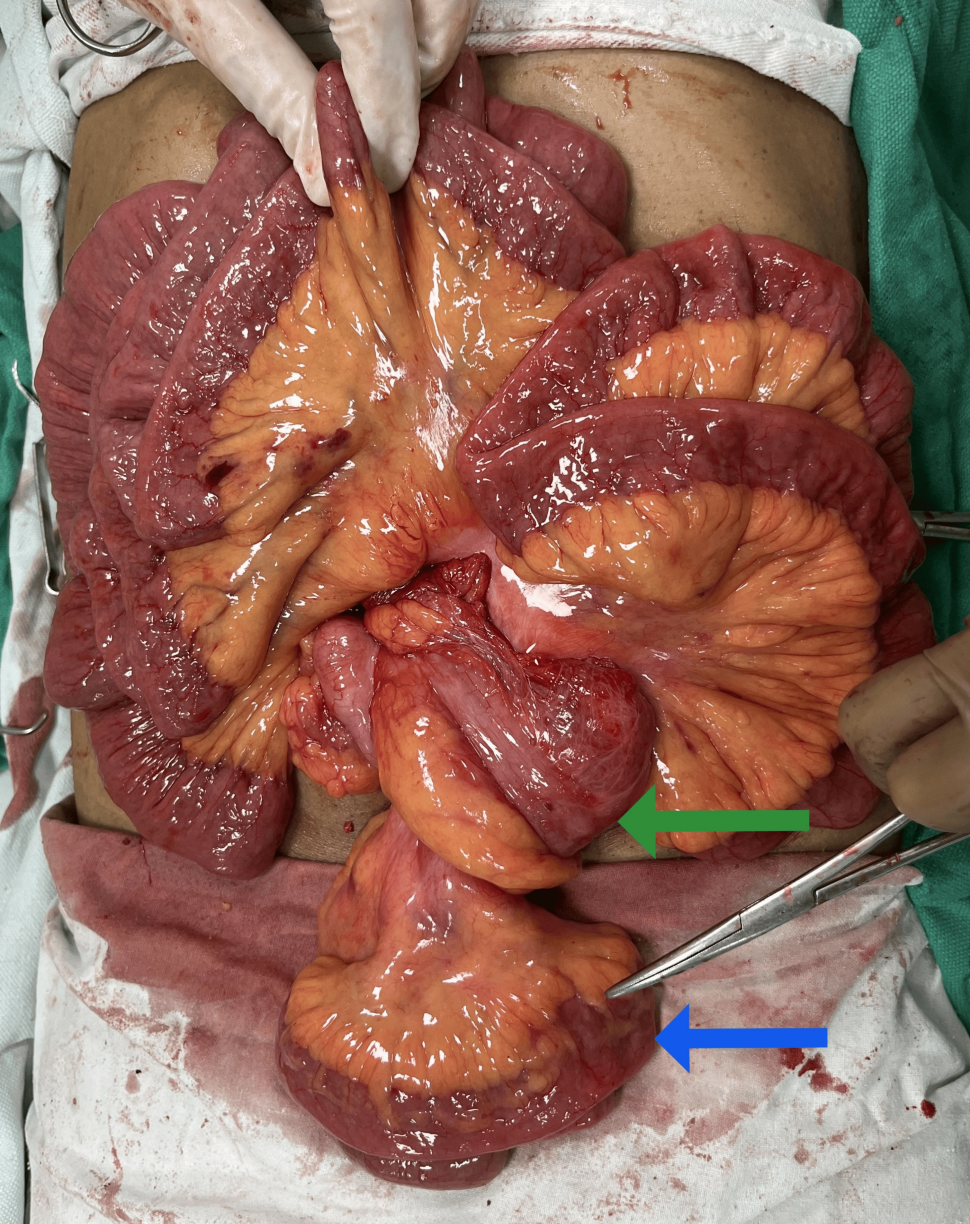
Intra-operative image after excision of sac showing freed small bowel and mobile caecum (green arrow) with ileal loops (blue arrow) that were already outside the sac

A diagnostic laparoscopy was done which showed a membrane covering the bowel loops. The extent of the sac could not be ascertained so the surgery was converted to exploratory laparotomy. The entire small bowel except 30 centimeters of distal ileum was found enclosed in a membranous sac (Figures 4-6). Mild adhesions between the anterior abdominal wall and sac were present. Flimsy inter-bowel adhesions were also seen. DJ flexure was seen on the right side of the midline and the caecum was very mobile. Adhesiolysis with the excision of the sac and caecopexy were done.

Histopathology examination of the membrane showed a fibrocollagenous membrane with a simple epithelium lining and no evidence of atypia, malignancy, or granuloma. GeneXpert of tissue was negative for tuberculosis. The patient had an uneventful recovery. He had no complaints on the one-year follow-up.

## Discussion

SEP can be either primary or secondary. Primary (idiopathic) SEP has also been termed abdominal cocoon syndrome. It is commonly seen in teenage girls in tropical and subtropical countries [2]. It was postulated that subclinical primary viral peritonitis due to immunological reaction to gynecological infection, retrograde menstruation, or retrograde infection of the fallopian tube is the etiology [5]. However, the etiopathogenesis in males, children, and perimenopausal women remains unknown. Idiopathic form of abdominal cocoon rarely occurs in male patients as seen in our case. Secondary SEP is more common with varying causes (Table 3) [5].

**Table 3:** Causes of secondary SEP SEP: sclerosing encapsulating peritonitis

	Causes of secondary SEP
1.	Peritoneal dialysis
2.	Abdominal tuberculosis
3.	Prior abdominal surgeries or trauma
4.	Recurrent bouts of peritonitis
5.	Autoimmune disorders like sarcoidosis and systemic lupus erythematosus
6.	Ventriculoperitoneal and peritoneovenous shunts
7.	Long-term use of beta-blockers (practolol) and methotrexate
8.	Toxins like asbestosis
9.	Liver cirrhosis and orthoptic liver transplant
10.	Gastrointestinal malignancy
11.	Intraperitoneal chemotherapy
12.	Familial Mediterranean fever
13.	Endometriosis, gynecological neoplasms, and dermoid cyst rupture
14.	Intra-operative abdomen wash given with povidone iodine

Peritoneal dialysis is the most common cause in developed countries whereas abdominal tuberculosis is more common in developing countries [6]. The percentage of patients developing SEP after peritoneal dialysis is 2.5% and incidence rises with duration [7]. Since our patient had no underlying cause, it was considered to be a primary cocoon.

Though the exact cause is unknown, it is hypothesized that peritoneal irritation incites inflammation that results in peritoneal fibrogenesis secondary to cytokine release and fibroblast activation [5]. The pathophysiology has been divided into four phases: presclerosing, inflammatory, progressive, and fibrotic [7].

The primary type can be associated with anatomical abnormalities like the absence of the omentum, visceral transposition, malrotation, cryptorchidism, and other conditions [8]. Our case presented a unique form of partial malrotation, not seen in the other cases published so far.

Based on the extent of encasement, abdominal cocoons are divided into three types. Type 1 is when part of the small intestine is enclosed. Type 2 is when the entire small intestine is enclosed. Type 3 is when along with the small intestine other organs like the appendix, caecum, ascending colon, ovaries, and other viscera are enclosed [9]. In our case, almost all small bowel loops except the distal ileum were enclosed so it was type 1.

Patients may remain asymptomatic for years. Some have non-specific symptoms like nausea, vomiting, loss of appetite, malnutrition, and recurrent abdominal pain while others present with recurrent attacks of subacute obstruction. Rarely, a soft and painless mass may be palpable [5]. At times, patients may present to emergency with acute abdomen. Initially, the capsule is thin so symptoms are less; but over time the capsule thickens and shortens resulting in obstruction. Our patient presented with features of subacute obstruction.

Sonography can show the "cauliflower" appearance of small bowel loops with a narrow base within a membrane in a concertina fashion. "Trilaminar sign" may be seen which consists of a superficial hyperechoic membrane, a middle hypoechoic layer of the bowel wall, and a deep hyperechoic layer of bowel gas [10].

CT is the gold standard in radiological investigation as it helps to differentiate from other causes of pain and obstruction. It shows small bowel loops conglomerated in the midline encircled by a non-enhancing soft tissue-dense membrane [1]. Occasionally, a definitive diagnosis is made intraoperatively. In our case, CT abdomen showed bowel loops encased by a peritoneal membrane.

Differential diagnosis with similar clinical and radiological features includes peritoneal tuberculosis; internal hernia (entry and exit point of bowel loops in sac should be close enough); pseudomyxoma peritonei; congenital peritoneal encapsulation (noninflammatory accessory peritoneal membrane found between mesocolon and omentum with entire small bowel posterior to it) [11,12].

Asymptomatic patients are kept on follow-up. Patients with mild symptoms can be managed conservatively with bowel rest, nasogastric decompression, and parenteral nutrition. Medical management can be considered when symptoms fail to regress. Anti-inflammatory or anti-fibrogenic drugs can be considered like colchicines, tamoxifen, steroids, azathioprine, and mycophenolate mofetil. Their use is advocated in secondary SEP but there is no data to support use in idiopathic cases. Relapse is known to occur [5].

Severe cases require surgery with excision of membrane and adhesiolysis. Laparoscopy can be diagnostic but therapeutic surgery can be challenging in advanced cases [5]. Bowel resection is needed only for ischemic or perforated bowel and is associated with increased mortality and morbidity. Over-aggressive surgery in patients with dense adhesions might result in iatrogenic perforation seen more often in cases of tuberculosis [8].

In our case, due to persistent symptoms, the decision to do surgery was taken. A laparoscopy was done to confirm the diagnosis which was then later converted to open surgery and an excision of the sac was done.

The most common postoperative complication is early postoperative small bowel obstruction. It usually develops within 30 days in those who have undergone extensive adhesiolysis with long operative time resulting in bowel edema [5].

Histopathological examination of the membrane reveals thickened and inflamed vascular fibrocollagenous tissue with infiltrating lymphocytes and plasma cells [13]. In our case, the histopathological examination showed similar features and did not show any granuloma or giant cells ruling out tuberculosis.

## Conclusions

SEP is a rare cause of intestinal obstruction and requires a high level of clinical suspicion to differentiate it from other usual causes of intestinal obstruction. SEP should be considered a differential in those with predisposing factors and chronic symptoms. Due to non-specific symptoms, it causes a delay in diagnosis. It may cause unexplained malnutrition and recurrent attacks of obstruction, for which surgery is indicated. Surgery was considered for our patient due to persistent symptoms and CT findings. For primary abdominal cocoon, surgery can be therapeutic without recurrence.
